# The Effectiveness of Home Services in Taiwan: A People-Centered Approach

**DOI:** 10.3390/ijerph15112605

**Published:** 2018-11-21

**Authors:** Li-Fan Liu, Wei-Ming Wang, Yi-Jung Chen

**Affiliations:** 1 Institute of Gerontology, College of Medicine, The National Cheng Kung University, Tainan 701, Taiwan; z10308108@email.ncku.edu.tw; 2 Department of Statistics, College of Management, The National Cheng Kung University, Tainan 701, Taiwan; r28051012@mail.ncku.edu.tw

**Keywords:** person-centered, health outcomes, latent class analysis, home and community-based service, long-term care (LTC)

## Abstract

Among available home and community-based services in Taiwan, there should be a focus on providing people with people-centered, value-based services. This study aims to follow up the people-centered health outcomes of care recipients and to examine the factors associated with to feedback for policy and practice in long-term care. A total of 9889 persons from the long-term care dataset were followed up for two years (T0–T4). The Cox Proportional Hazard Regression analyses to analyze mortality and the mixed effect models for health outcomes were used. Three classes among the care recipients were identified. Health profiles (HR = 1.46 and 2.56 for FI and FD compared with RI, *p* < 0.001), subsidy gap (HR = 1.01), and living status (HR = 0.88 for those living with spouse only) had a significant impact on mortality. The overall dropout rate was 52.3% at two years, and the health profiles at baseline significantly influenced the health outcome’s change. The health heterogeneity matters and influences subsequent outcomes. To reach the goal of the HCBS, regular and exact monitoring of care recipients is crucial, while feedback regarding health outcomes and a greater focus on providing person-centered and responsive services in the community are also required.

## 1. Introduction

For people living with complex long-term conditions, a trend towards value-based health services, striving to cut costs while generating value, has been observed [1]. An effort to achieve value-based health services that provide the health outcomes that matter to patients and providing care that is more patient-centered will place increased demands on the healthcare system [2]. Healthcare has shifted from disease-oriented to integrated patient-centered medicine [3]. An effort is being made to focus medical attention on the individual patient’s needs and concerns, rather than on those of doctors. People-centered service delivery has also been emphasized by WHO [4]. The growing demands for quality and safety in health care have refocused attention on patient outcomes [3]. In the process, integrating knowledge and evidence-based approaches to ageing in national health policy development will be essential to maintain active ageing societies. Understanding how to make use of evidence to develop a national approach to policy should be equally important to service delivery in order to benefit care recipients and the aging society as a whole [5]. Among the goals of the health care system, the goal of effectiveness (quality), which relates to health outcomes, does involve what benefits are obtained and who obtains them in the system [6]. Therefore, building evidence-informed policies to respond to the health needs of an ageing population is essential.

In Taiwan, with the trend of population aging, the need for long-term care is increasing rapidly. At the end of 2017, 14% of the population of Taiwan was aged 65 years and over, and it is estimated that the elderly population will triple to 43% by 2061 [7]. Due to the multiple comorbid chronic conditions and complex care needs of older people, it is estimated that among those who need long-term care, nearly 75% are older Taiwanese aged 65 and over [8].

Taiwan has had a well-established National Health Insurance system to cover people’s needs for acute care since 1995. In planning for provision of the country’s long-term care (LTC), the Taiwanese government launched the LTC 1.0 policy in 2007, aimed at helping frail elderly people with LTC needs. To facilitate service delivery and reform LTC policies, the government began transitioning to the development of a new LTC 2.0 policy as of 2016 and intended to cover all people with LTC needs in Taiwan [9]. Apart from institutional care, the LTC 1.0 policy was aimed at developing the home and community-based services (HCBS), including home services, adult day care, home nursing care, home and community-based rehabilitation, home meal delivery, palliative care for caregivers, and transportation services. Among them, home services are the most common type of service that is used long-term [9,10]. Based on household social welfare status, LTC recipients have co-pay service charges, which was 30% for non-low and 10% for mid-low income households in the 1.0 policy. These financial subsidies are mainly provided by the central government. Since financing the LTC service delivery is based on tax payments, it is currently separated from the National Health Insurance, which covers the acute care payment system. However, because the goal is to achieve a continuum of care, more efforts, such as linkage to discharge plans accompanying LTC care management and service delivery has been added in the changes reflected in the LTC 2.0 policy.

A good understanding of the health heterogeneity of elderly people, their characteristics, patterns of health care utilization and subsequent outcomes and expenditures is necessary to evaluate adequately the policy options and interventions aimed at improving the quality and efficiency of care for older people. Although the national long-term care system in Taiwan has been used to overcome the fragmentation resulting from the decentralization of the LTC system, there are few evaluations of it. Needs assessments are one of the most important core tasks of care managers, as they determine clients’ subsequent care plans by using the information obtained during the initial assessment. Although the care recipients need to be reassessed every six months under government regulations, the results are not necessarily analyzed, and, therefore, there is a lack of information regarding the outcome changes. Examining the health outcomes of care recipients is a critical step in planning and quality improvement, and it is also useful to measure performance at different levels [11].

As Kruk and Freedman [12] noted, evaluations of the effectiveness of health care systems tend to focus on health status and patient satisfaction (outcomes), as well as on access and quality of care (outputs). In view of the rapid expansion of the LTC service network and increasing use of such services in Taiwan, it is important to obtain information on the changing health status of care recipients by following up their health outcomes. In order to continue and improve the HCBS, it is important to measure the results according to people-centered outcomes that matter to care recipients and provide feedback on the effects of the current service provision and policymaking.

Previous studies have shown that “universal” outcome measures, such as general health, the activities of daily living (ADLs), the instrumental activities of daily living (IADLs), cognitive status, depression, resident satisfaction, and quality of life, are needed across diseases and that outcome measures can be applied to improve quality and determine payments [13,14,15,16]. Therefore, apart from the physical functions, indicators regarding mental health are also important, since it is evident that psychosocial interventions such as social activities have positive effects on quality of life, mental health and reduction of depressive symptoms [17,18]. For care recipients in the HCBS, this research is aimed toward an exploration of care recipients’ health outcomes and an examination of the factors associated with receiving home service over a two-year period. To the best of our knowledge, this is the first study examining the health outcomes of care recipients and providing follow-up. It is hoped that the outcome evaluations will not only add information about the care recipients, but also prove useful information with regard to making effective improvements to the practices in LTC.

## 2. Materials and Methods

### 2.1. Data Source and Study Design

The data was obtained from the long-term care dataset (LTC-CM) acquired by the Taiwanese government. The dataset has been maintained by the LTC center in each county since 2008, in which the health indicators of care recipients are recorded in an initial need assessment and reassessed by care managers during the follow-up process. The data from each local government is uploaded to the Ministry of Health and Welfare for the purpose of financial reimbursement from the central government. The data set from some local governments has been used in some previous studies [19].

The study is therefore a retrospective design used to analyze records in the long-term care dataset from 2011–2015 to examine the changes in outcomes and the factors associated with receiving home services.

### 2.2. Settings and Participants

In this study, we analyzed the long-term care dataset of one southern city in Taiwan. The sample city is one of the six metropolitan areas in Taiwan and the LTC service delivery is identical under the government LTC policy. LTC recipients who receive HCBS, noted above, are recorded in the dataset.

When data for the present study was firstly collected in 2016, a total of 10,450 care recipients aged 50 and over had initial needs assessment (T0) records from 2011–2015 kept in the dataset. After data cleaning, a total of 9889 persons had records for the initial need assessments (T0) with home service subsidies. Among these, 6137 had one reassessment at six months (T1), 3490 persons had records for reassessments through one year (T2), 2214 for one and half years (T3), 1398 for two years (T4), and 848 for T5 and above. For the analyses of outcome changes, we included primary respondents who did not respond through a proxy and remained in the HCBS for a maximum of two years from the baseline.

### 2.3. Measures

Four main outcome measures, including the activities of daily living (ADLs), instrumental activities of daily living (IADLs), the Short Portable Mental Status Questionnaire (SPMSQ) and the short version of the Centre for Epidemiologic Studies Depression Scale (CESD). The ADLs scores 0–30; 31–60; 61–100; the IADLs scores 0–8, 9–12 and the higher scores the better physical function ability. The SPMSQ in the need assessment questionnaire, scores of 1 to 4 ranging from severe cognitive impairment to no cognitive problem, were assigned after adjusting for the educational levels assessed by care managers, and the CESD; scores ≥ 12 for males and ≥ 10 for females, indicating depressive tendency, were used in the assessment questionnaire.

### 2.4. Data Analyses

The data analyses for the study included descriptive and inferential statistics. A latent class analysis (LCA), a model-based tool suitable to identify heterogeneity, was firstly conducted to group individuals into categories in order to identify the health profiles of care recipients, each of which contains care recipients who are similar to each other and different from individuals in other categories [20]. The Cox proportional hazard regression analysis and the mixed effect models were used to analyze the predictors for mortality and four follow-up outcome variables. All analyses were performed using Mplus 7.0 (Muthén & Muthén, Los Angeles, CA, USA) and SAS version 9.4 (SAS Institute Inc., Cary, NC, USA). The study protocol (A-ER-104-384) was approved by the National Cheng Kung University Human Research Ethics Committee, and no conflicts interest existed between the authors and the goals of this study.

## 3. Results

### 3.1. Health Profiles of Care Recipients at Baseline (T0) and Their Characteristics

For LCA, the health indicators chosen were mainly the four universal health measures shown in Table 1. These indicators were related to need factors as measured by functional impairments as the potential endogenous factors that influenced the long-term care needs of elderly people. There were three classes identified with the best model fit (LMR-LRT, *p* < 0.001; Entropy = 0.946). A label was assigned to each class based on comparisons of conditional item response probabilities. The first group was the “Relatively Independent” (RI) group. This group of care recipients was comparatively healthy and had relatively few functional limitations with no or least difficulties for both ADLs (*λ* = 0.005 with score 0–30) and IADLs (*λ* = 0.000 with score 0–8) and high probability of no cognitive problem (*λ* = 0.834) and no depression tendency (*λ* = 0.929). The second was the “Functional Impairment” (FI) group with relatively high probabilities of mild difficulties (*λ* = 0.910 in ADLs 61 + and *λ* = 1.000 in IADLs with score 0–8), less than half with cognitive problems (*λ* = 0.483) and only a few with depressive symptom (*λ* = 0.098). In the third group, individuals had the highest probabilities of both physical (with high difficulties in ADLs score 0–30 and IADLs score 0–8, *λ* = 0.915 and *λ* = 0.983, respectively) and cognitive impairment (*λ* = 0.282 with no cog. impairment), and a higher depressive tendency (*λ* = 0.126) when compared with the other two groups, labeled as “Frail and Dependent” (FD). The three classes of elderly people in the study included 3249 (33%) people in the RI group, 4440 (45%) in the FI group, and 2200 (22%) in the FD group.

In terms of the characteristics of care recipients, Table 2 shows the details. As for health status, it is obvious that the care recipients in RI group were those with better ADLs (80.87 ± 13.03), IADLs (12.36 ± 3.13), SPMSQ (3.76 ± 0.59) and CESD (6.45 ± 2.60) scores. All the characteristics listed in Table 2 (including age, gender, living status, household social welfare status, health status and the caregivers’ characteristics) were significantly different, meaning that there are existing differences among the three group of care recipients.

### 3.2. Two-Year Mortalities

It was found that the two-year survival rate of care recipients is 75.9%, with the highest survival rate (86%) in RI and the lowest in FD (59.5%) group. Table 3 shows the potential risk factors for mortality in two years. We found that the health profiles had a significant impact on mortality (HR = 1.46 and 2.56 for FI and FD compared with RI, *p* < 0.001), as did the subsidy gap (HR = 1.01, *p* < 0.001) and living status (HR = 0.88 for those living with spouse only, *p* < 0.05), apart from the influence of age and gender among the long-term care recipients.

### 3.3. The Outcome Changes of Care Recipients from t0–t4 and the Predicting Factors

Apart from death, the overall dropout rate was 52.3% at two years (T4), meaning that nearly half of the care recipients chose to leave the service in two years. In this study, the predictors of health outcome changes were examined using mixed effect models for the outcomes up to the maximum of two years. Table 4 shows the details.

(a). The Different Health Profiles at T0 Influenced the Health Outcome Changes

The results showed that the health profiles examined at T0 significantly influenced the health outcome changes. When using the RI group (RI) as a reference, it is clear that the outcome variables of ADLs, IADLs and SPMSQ, the FI and FD group, were more likely to have worse outcome changes with negative estimates (for ADLs: *β* = −7.070 for FI, −49.523 for FD, *p* < 0.001; for IADLs: *β* = −6.362 for FI, −9.26 for FD, *p* < 0.001; for SPMSQ: *β* = −0.667 for FI, *p* < 0.01; −1.452 for FD, *p* < 0.001). It meant that the lower the scores, the worse the outcome changes. The CESD scores also showed worse outcome changes in the FD group with positive estimates (for CESD: *β* = 0.606 for FD, *p* < 0.001) since the higher the CESD scores, the worse the outcomes.

(b). The Outcome Changes from T0 to T4

The results showed that the outcomes change differently with different health profiles. When controlling for all other factors, the scores of ADLs increased for the FD group (*β* = 1.347 for FD x time, *p* < 0.001), while the outcome change of FI decreased (*β* = −0.531, *p* < 0.05) when compared with RI x time (Table 4). Regarding the scores of IADLs, these increased significantly from six months (T1) to the two-year follow-up time point (T4) (*β* = 0.169 for FI x time, *p* < 0.001, *β* = 0.213 for FD x time, *p* = 0.001). However, the SPMSQ and CESD outcomes showed no significant changes.

(c). The Influences of Socio-Demographic Factors

Some socio-demographic factors were found significant when compared with the reference group of each variable, as shown in Table 4. In general, age, gender, living status, education level, and household social welfare status were significantly associated with changes in the scores for the outcome variables. For example, the educational level of the illiterate was negatively associated with changes of the IADLs (*β* = −0.533, *p* < 0.001), SPMSQ (*β* = −0.180, *p* < 0.001) and CESD outcome scores (*β* = 0.276, *p* < 0.001). Living alone showed a positive relationship with ADLs (*β* = 7.589, *p* < 0.001), IADLs (*β* = 1.719, *p* < 0.001), and SPMSQ scores (*β* = 0.178, *p* < 0.001).

It was also found that the household social welfare status was a predictor of the care recipients’ outcomes. For example, when comparing those with non-low income households, those mainly from mid-low/low incomes (getting social welfare subsidies) were more likely to have negative estimates on the change in scores of the ADLs and CESD (*β* = −2.316, *p* < 0.001 and *β* = 0.150, *p* < 0.05, respectively), meaning that worse outcomes among them. Moreover, caregiver burden significantly influences the outcomes of ADLs, IADLs and CESD (*β* = −0.995, −0.275 and 0.487, respectively, *p* < 0.001). Finally, the subsidy gaps also were found to have a significant influence on the outcome changes in ADLs and IADLs (*β* = −0.267, *p* < 0.001 and *β* = −0.003, *p* < 0.05, respectively) in this study.

## 4. Discussion

A common criticism of home and community services mentioned previously is that they are fragmented, resulting in poor outcomes and wasted resources [21]. In considering the development of community care, to what extent the related initiatives lead to better outcomes in terms of meeting unmet needs matters [22]. Although the government conducts regular accreditations, cross-sectional inputs and outputs remain the focus in Taiwan. This study explored the changes in outcome and examined the factors associated with receiving home services over a two-year period. However, long-term care recipients can also have different health outcomes influenced by their health and functional heterogeneity. Some key findings in the results that require further attention are discussed as follows:

### 4.1. Health Heterogeneity Matters When Choosing an HCBS

Among the HCBS in Taiwan, home services are used more often and on a long-term base than other services. It may be that the service is more convenient, such as delivering to people’s own homes. It has been firstly rooted in the community since 1992. However, this study showed that the two-year outcomes of care recipients were significantly influenced by their health and function profiles at baseline. The health profiles of the FI (FI) and FD (FD) groups at baseline had worse outcomes, including mortality, on all four outcome indicators when comparing with RI. Although previous research suggested that all-cause mortality rates are higher among adults with disabilities than among adults without disabilities [23], the finding in our study still leads to concern about the care adequacy for those with relatively severe dependency. Is the delivery of home services alone a way to maintain their health and functional status? Should more assistive technology (AT) devices be included in home services apart from the manpower of care assistants? As indicated in previous research [24], assistive technology devices can be divided into five categories, including a barrier free environment (home modifications), daily living aids, mobility aids, seating and positioning devices, and sensory aids. Proper application of AT devices can help achieve the goal of aging in place. There is a need to address in future research if other inputs or options are needed for people with severe dependency on home services. Previous research has shown that case management improves function and increases use of community services; however, the results for the clinical outcomes were not consistent, or the factors under examination had little effect on the clinical outcomes [25]. Other outcome measures could also be included in the follow-up research such as client satisfaction and family caregiver burden [18,26]. It is critical to follow-up the care outcomes instead of presuming services delivered to homes can be one service fits all.

### 4.2. The Outcome Changes of Different Health Profiles

In this study, the health indicators of ADLs and IADLs showed positive outcome changes among the care recipients. More specifically, the ADLs scores increased for the FD group with increasing time, and the IADL scores increased for the FI and FD groups when comparing with RI. This could be due to the low average ADLs score (11.73 ± 10.88) in FD at baseline and therefore make larger room for improvements after home service intervention. Physical health is a significant predictor of cognitive impairment in elderly people [27]. Access to basic amenities in the home was found to be the first key determinant of quality of life and self-esteem among Asian disabled people [28]. With increasing time, the significant improvements in IADLs score were found in the study and difficulties with IADLs were shown to have the highest prevalence of unmet/under met needs for long-term care [29]. Thus, the improvement of physical functions serves as an important outcome measure to match the needs of those with difficulties in daily life. However, in considering the high dropout rates, the outcome changes were analyzed for those remaining in the service, and there were no follow-up data of those who dropped out to compare with.

As for the mental health outcomes, SPMSQ showed no significant changes, so is the health outcomes of CESD with increasing time. The results remind us that long-term care should be a means to ensure that old people with a significant loss of capacity can still experience healthy aging by optimizing the care recipients’ intrinsic capacity and by providing the environment support and care necessary to maintain functional ability at a level that ensures well-being [4]. It is apparent that less psychological support is made available than would be ideal. Current services in Taiwan seem more focused on vulnerable care recipients with difficulties in physical abilities, as well as those who were single, living alone, and/or living in low-income households, since these were the characteristics more easily detected than complicated multiple needs factors, especially psychological ones [19]. The importance of psychological support would imply the need for more innovative home services or integrating its delivery to people’s own homes. For the LTC policy in Taiwan, there remains a need for more research from the provision of professional consultations and help for LTC recipients.

Regarding the follow-up outcome changes, our findings were in accord with previous research that the majority of long-term care recipients revealed a similar global cognitive decline rate [30,31]. However, comparing to the estimates of different health profiles at baseline, it is evident that the different health profiles of care recipients need to be noticed and taken into account since it had much high impact on health outcomes. Also the adequacy of care for the care recipients of different health profiles with different level of dependency needs to be further addressed.

### 4.3. The Characteristics of Care Recipients and Rethinking About the Caregiver Burden and the Subsidy Gap

It is not surprising that old age has been associated with life course functional decline and cognitive impairments [32,33]. Gender influence in outcomes is in line with the findings of previous research [34,35]. Those with lower educational levels as well as lower income have also been found to have worse outcomes, which is consistent with the findings of previous research indicating that worse outcomes are found among those with disadvantages [36,37]. In fact, the LTC policy in Taiwan is a social welfare policy, and the program aims to take care of more disadvantaged elderly people. The care recipients in HCBS have, thus, been selected by the care management system. In addition, the caregivers’ burden showed a significant influence on health outcomes, which is consistent with previous research [25], since the need is for family caregivers to standby and take care of their dependent relatives in their own homes.

Meanwhile, the subsidy gaps showed a significant impact on outcomes reflecting the possibility of having not enough care assistance or unmet needs. Since the overall dropout rate is high, it seems that the care recipients and their families did not choose to stay put in receiving home service. Previous research regarding HCBS showed that greater unmet needs predicted worse outcomes, such as nursing home placement, death, and loss to follow-up [26,38], and people who report unmet needs tend to be in worse health and with lower income [39]. This study found that the current lower level of subsidies provided for formal home services influenced health outcomes, and it remains unclear whether their utilization behaviors are among other reasons. However, a lack of sufficient care can have negative consequences when a person is in need of long-term care services, which may compromise a person’s safety in the community and impede the management of health problems [40].

Previous research showed that the concept of unmet need includes accessibility, availability or adequacy of care assistance [40]. The LTC policy in Taiwan with universal coverage is aimed at providing HCBS on a nationwide scale with better accessibility. However, the availability of service resources, such as workforce and financial subsidies, and also the adequacy of care for people with various functional statuses, should be as important as getting access to LTC services in the community. These problems/issues could also be reflected in the high dropout rates and deserve more attention in LTC policy making and innovative resource allocation.

As mentioned in previous research, Asian countries have not reported evidence for compression of morbidity [41]. Under the goals of the LTC policy in Taiwan, there is a necessity to continue to develop and provide HCBS, which could match LTC needs with good quality services. Since the service delivery of HCBS in the LTC system is based on the care management system in Taiwan, it is also important how the care managers function as coordinators to deliver services and monitors for after needs assessment and implementation of care plans, as such steps are critical to obtaining the feedback needed to improve service delivery. The relevant outcome measures with a linked reimbursement system are supposed to drive development towards higher quality in the upcoming emphasis of value-based health services. This shift has the potential to align healthcare-service delivery with person-centered care [1]. This research used LCA methodology to identify the health heterogeneity of care recipients. It is hoped that the LTC needs and outcome changes resulting from the use of a person-centered approach will add information about who benefits and how such benefits change after receiving care. Although deterioration among care recipients will inevitably be found, it is still critical to monitor the health outcomes of care recipients in order to determine factors that can provide information related to quality-improvement initiatives in various contexts.

### 4.4. Limitations

This study has the following limitations. Firstly, only long-term care recipients receiving home and community-based care/services were reported and recorded in the LTC dataset. Secondly, in practice the health outcomes of care recipients are also influenced by caregiving in the family network, such as being taken care of by family members and/or informal paid help, since, in Taiwan, families remain crucial support networks and elder care remains a family responsibility. Thirdly, the follow-up outcomes were chosen based on the available health measurements, including physical and mental outcomes, whereas outcomes regarding social health were not included, since they were not available in the dataset. In addition, since the data set used was on the needs assessment of LTC recipients, information related to the perspectives from care recipients and caregivers were not included. Finally, the nationwide LTC dataset has yet to be formally released and the samples used in this study came from the LTC dataset of one southern city in Taiwan, thus, the generalization of these findings should be handled with caution when considering geographical differences, LTC service resources, and supply.

## 5. Conclusions

The shortage of research about the effectiveness of interventions has hindered the development of appropriate evidence-based policies and practice in long-term care. In viewing the importance of health outcomes, this research examined the health profiles of care recipients and found patterns of changes in outcomes. The results showed that health heterogeneity matters and significantly influences the outcomes. In addition, while the changes of physical functions were found to have better outcomes with various dependency levels, mental health outcomes were found to have no significant difference. To reach the goal of HCBS, feedback with regard to health outcomes and a greater focus on providing person-centered and responsive services in the community is required. In the ongoing process, such efforts would provide not only care information regarding the care recipients, but it would also allow for more informed decisions toward value-based health services in policymaking.

## Figures and Tables

**Table 1:** Health profiles of the LTC recipients by using Latent Class Analysis.

Variable	HP *					
	RI (*N* = 3249)		FI (*N* = 4440)		FD (*N* = 2200)	
	Prob.	*p*-Value	Prob.	*p*-Value	Prob.	*p*-Value
ADL						
0–30	0.005	0.006	0.000	1.000	0.915	0.000
31–60	0.085	0.000	0.386	0.000	0.085	0.000
61+	0.910	0.000	0.614	0.000	0.000	1.000
IADL						
0–8	0.000	1.000	1.000	1.000	0.983	0.000
9+	1.000	1.000	0.000	1.000	0.017	0.000
Cognition status						
Severe	0.010	0.000	0.080	0.000	0.459	0.000
Moderate	0.051	0.000	0.236	0.000	0.147	0.000
Mild	0.105	0.000	0.201	0.000	0.111	0.000
Normal	0.834	0.000	0.483	0.000	0.282	0.000
Depression						
No	0.929	0.000	0.902	0.000	0.874	0.000
Yes	0.071	0.000	0.098	0.000	0.126	0.000

Note: 1. *N* = 9889 for LTC recipients with the baseline and at least one reassessment afterwards; 2. HP *: Health Profiles of the LTC recipients at baseline (RI, FI, and FD); Prob. denotes Probabilities. 3. The LTC dataset from a metropolitan city in 2011–2015. 4. The categorical cuts for the outcome variables: a. ADLs score 0–30; 31–60; 61–100. The higher scores the better physical function ability. b. IADLs score 0–8; 9–12. c. Cognition status was categorized in MDAI as 1–4, ranging from severe cognitive impairment (1) to no cognitive problem (4). d. CESD: Scores were ranging from 0–20. The higher scores, the worse condition of depressive tendency. The thresholds of depressive tendency (coding: 1): male ≥ 12, female ≥ 10 according to the standard in the need assessment of LTC and no depressive tendency: 0 (as the reference group).

**Table 2:** Socio-demographic characteristics and health status of the three profiles of care recipients in the Long-Term Care Dataset at baseline.

	RI (*N* = 3249)	FI (*N* = 4440)	FD (*N* = 2200)	*p*-Value
Gender				<0.001
Male	1248 (38.4)	1812 (40.8)	1050 (47.7)	
Female	2001 (61.6)	2628 (59.2)	1150 (52.3)	
Age	79.04 ± 10.40	82.02 ± 9.47	79.86 ± 10.76	<0.001
50–75	931 (28.7)	863 (19.4)	625 (28.4)	<0.001
75–85	1286 (39.6)	1713 (38.6)	805 (36.6)	
85+	1032 (31.8)	1864 (42.0)	770 (35.0)	
Educational levels				<0.001
Illiterate	1457 (44.8)	2522 (56.8)	1156 (52.6)	
Elementary school	1016 (31.3)	1202 (27.1)	626 (28.5)	
Junior high school	314 (9.7)	251 (5.7)	182 (8.3)	
Senior high school	278 (8.6)	268 (6.0)	146 (6.6)	
College	133 (4.1)	144 (3.2)	70 (3.2)	
Above degree	13 (0.4)	12 (0.3)	5 (0.2)	
Others	38 (1.2)	41 (0.9)	15 (0.7)	
Living Status				<0.001
With children	686 (21.1)	1582 (35.6)	807 (36.7)	
With spouse only	751 (23.1)	1130 (25.5)	499 (22.7)	
Living alone	1271 (39.1)	606 (13.7)	53 (2.4)	
With spouse and children	335 (10.3)	834 (18.8)	702 (31.9)	
Living with grandchildren or relatives	169 (5.2)	248 (5.6)	110 (5.0)	
Others	37 (1.4)	40 (0.9)	29 (1.3)	
Social Welfare Status				<0.001
Non-low Income households	2634 (81.1)	3826 (86.2)	1794 (81.6)	
Mid-low income households	291 (9.0)	287 (6.5)	246 (11.2)	
Low income households	324 (10.0)	327 (7.4)	160 (7.3)	
Caregivers’ Characteristics				<0.001
Full-time caregiver	663 (20.4)	1800 (40.5)	1410 (64.1)	
Part-time caregiver	1542 (47.5)	2407 (54.2)	765 (34.8)	
Others	1044 (32.1)	233 (5.3)	25 (1.1)	
Caregiver burdens (total scores)	4.06 ± 1.11	4.48 ± 1.19	4.68 ± 1.19	<0.001
Health status (scores)				
ADLs	80.87 ± 13.03	65.66 ± 16.57	11.73 ± 10.88	<0.001
IADLs	12.36 ± 3.13	4.89 ± 2.21	1.92 ± 2.73	<0.001
SPMSQ	3.76 ± 0.59	3.05 ± 1.05	2.21 ± 1.28	<0.001
CESD	6.45 ± 2.60	6.97 ± 2.45	7.56 ± 2.54	<0.001
Subsidy gap # (hours)	15.97 ± 17.70	33.90 ± 25.41	60.46 ± 15.96	<0.001
Living Area				<0.001
Urban/sub-urban area	2419 (74.5)	3159 (71.2)	1544 (70.2)	
Remote area	830 (25.5)	1281 (28.9)	656 (29.8)	

Note: 1. *N* = 9889. 2. # the subsidy gap per month was calculated by the standard subsidy hours each dependency level listed deduced the actual subsidies issued by care managers to each care recipient in practice.

**Table 3:** The potential risk factors for mortality in two years.

Risk Factor	HR	*p*-Value
Male (ref. female)	1.53	<0.001 ***
Age 50–75 (ref. 85+)	0.68	<0.001 ***
Age 75–85 (ref. 85+)	0.80	<0.001 ***
Illiterate (ref. Literate)	1.01	0.811
Living with spouse only (ref. living with others)	0.88	0.041 *
Living alone (ref. living with others)	0.85	0.060
Mid-low/low incomes (ref. non-low incomes)	1.05	0.504
Caregivers’ burden	0.99	0.634
Subsidy gap	1.01	<0.001 ***
Urban/sub-urban area (vs remote area)	1.25	<0.001 ***
HP * FI (vs RI)	1.46	<0.001 ***
HP * FD (vs RI)	2.56	<0.001 ***
Wald Chi-Square	550.50	
AIC	28,752.58	

Note: 1. *N* = 9889. 2. HP *: Health Profiles of the LTC recipients at baseline. 3. Cox proportional hazard Regression (Deaths Event = 1642 deaths in two years). * *p* < 0.05; *** *p* < 0.001.

**Table 4:** Predictors of the two-year outcomes among the long-term care recipients in HCBS (Mixed effect model).

Fixed Effects	ADL	IADL	SPMSQ	CESD
	(*N* = 6137) (Observations = 13,237)	(*N* = 6137) (Observations = 13,239)	(*N* = 6137) (Observations = 13,239)	(*N* = 5197) (Observations = 11,210)
	Estimated Mean Difference (Std. Error)			
Male (ref. female)	−0.012 (0.343)	−0.179 (0.064) **	−0.051 (0.020) *	−0.195 (0.052) ***
50–75 (vs 85+)	1.392 (0.464) **	0.812 (0.083) ***	0.285 (0.026) ***	0.372 (0.067) ***
75–85 (vs 85+)	1.004 (0.360) **	0.472 (0.064) ***	0.144 (0.020) ***	0.313 (0.051) ***
Illiterate (ref. Literate)	−0.260 (0.352)	−0.533 (0.063) ***	−0.180 (0.020) ***	0.276 (0.051)
Living with spouse only (ref. living with others)	2.547 (0.393) ***	0.438 (0.070) ***	0.164 (0.022) ***	0.220 (0.057) ***
Living alone (ref. living with others)	7.589 (0.416) ***	1.719 (0.074) ***	0.178 (0.023) ***	0.205 (0.057)
Mid-low/low incomes (ref. non−low incomes)	−2.316 (0.407) ***	−0.001 (0.073)	−0.044 (0.023)	0.150 (0.059) *
HP * FI (vs RI)	−7.070 (0.576) ***	−6.362 (0.103) ***	−0.667 (0.032) ***	0.117 (0.080)
HP * FD (vs RI)	−49.523 (0.852) ***	−9.26 (0.152) ***	−1.452 (0.047) ***	0.606 (0.143) ***
FI x time (vs RI x time)	−0.531 (0.227) *	0.169 (0.040) ***	−0.001 (0.013)	0.000 (0.032)
FD x time (vs RI x time)	1.347 (0.347) ***	0.213 (0.062) ***	0.005 (0.019)	−0.048 (0.062)
Caregivers’ burden	−0.995 (0.132) ***	−0.275 (0.024) ***	0.006 (0.007)	0.487 (0.019) ***
Subsidy gap	−0.267 (0.007) ***	−0.003 (0.001) *	0.000 (0.000)	0.001 (0.001)
Urban/sub-urban area (vs remote area)	−0.850 (0.351) *	−0.036 (0.063)	0.086 (0.020) ***	0.389 (0.050) ***

Note: 1. *N* = 9889. 2. * *p* < 0.05; ** *p* < 0.01; *** *p* < 0.001.
